# Importance of a vertically tilting structure for energizing the North Atlantic Oscillation

**DOI:** 10.1038/s41598-020-69551-5

**Published:** 2020-07-29

**Authors:** Patrick Martineau, Hisashi Nakamura, Yu Kosaka, Ayako Yamamoto

**Affiliations:** 1 Research Center for Advanced Science and Technology, University of Tokyo, Tokyo, Japan; 2 Japan Agency for Marine-Earth Science and Technology, Yokohama, Japan

**Keywords:** Atmospheric science, Atmospheric dynamics, Atmospheric dynamics

## Abstract

The North Atlantic Oscillation (NAO) is a prominent mode of atmospheric variability that influences weather and climate, including the occurrence of extreme events, over a large part of Europe and Northeastern America. The NAO has been considered to be maintained primarily by migratory weather disturbances and to have a deep structure with no vertical tilt. A careful inspection nonetheless reveals that the associated anomalies do exhibit a subtle vertical tilt, but its dynamical implications are still unknown. Here we show that this vertical tilt is of vital dynamical significance for the wintertime NAO. We find, using atmospheric reanalysis data, that the tilted anomalies transport heat across the pronounced thermal gradient associated with a background westerly jetstream, advecting air from the cooler North America and Greenland to the warmer Atlantic, thereby acting to reinforce NAO’s thermal anomalies. The resultant conversion of potential energy from the background state is a larger energy source for maintaining the NAO than the feedback from migratory disturbances. Our findings thus uncover a fundamental mechanism of the NAO dynamics, with implications for the improvement of seasonal predictions for the Euro-Atlantic climate and the representation of the NAO variability in climate models.

## Introduction

The North Atlantic Oscillation (NAO) is one of the most dominant modes of variability of the atmospheric circulation in the extratropical Northern Hemisphere^1–3^. It is responsible for an important fraction of surface weather fluctuations^4^ over the Euro-Atlantic sector, including Northeastern America and portions of Eurasia, modulating the occurrence of weather extremes^5–8^. Fundamentally, the NAO represents the meridional wobbling of the North Atlantic eddy-driven jetstream, whose poleward deflection tends to be stronger during the positive phase. The NAO also exerts an important influence on the underlying ocean^9^, including its physical^10^ and biochemical^11^ properties, as well as marine ecosystems^12^. Because the NAO-related weather and climate variability has such profound socioeconomic impacts as above, dynamical processes responsible for the formation and maintenance of the associated circulation anomalies have received considerable attention over the last decades.

The NAO is influenced by the state of the North Atlantic Ocean on interannual^13^ to multi-decadal^14,15^ timescales. Inter-basin teleconnections affecting the NAO are also observed between the North Pacific and North Atlantic sectors^16–19^. On intraseasonal timescales, the NAO is known to be influenced by the strength of the stratospheric polar vortex^20,21^. Most of the intraseasonal NAO variability is, however, driven and maintained in situ by quasi-stationary waves and high-frequency migratory cyclones and anticyclones^22–27^. This driving of the NAO by transient eddies manifests itself synoptically as wave breaking^19,28–32^, with a poleward shift of the jetstream occurring under anticyclonic breaking and the opposing shift under cyclonic breaking.

The forcing of the NAO by wave breaking is achieved by baroclinic (i.e., vertically tilted) eddies morphing into a more barotropic (i.e., vertically coherent) structure as they mature^27,31,33^, thereby imparting their barotropic momentum and energy signatures to the NAO. Because of this *apparent* lack of vertical tilt, little attention has been paid to the role of baroclinic processes arising from the NAO’s subtle vertical tilt^23^ in the formation and maintenance of its circulation and thermal anomalies.

## Results

### Three-dimensional structure of the NAO

**Figure 1:**
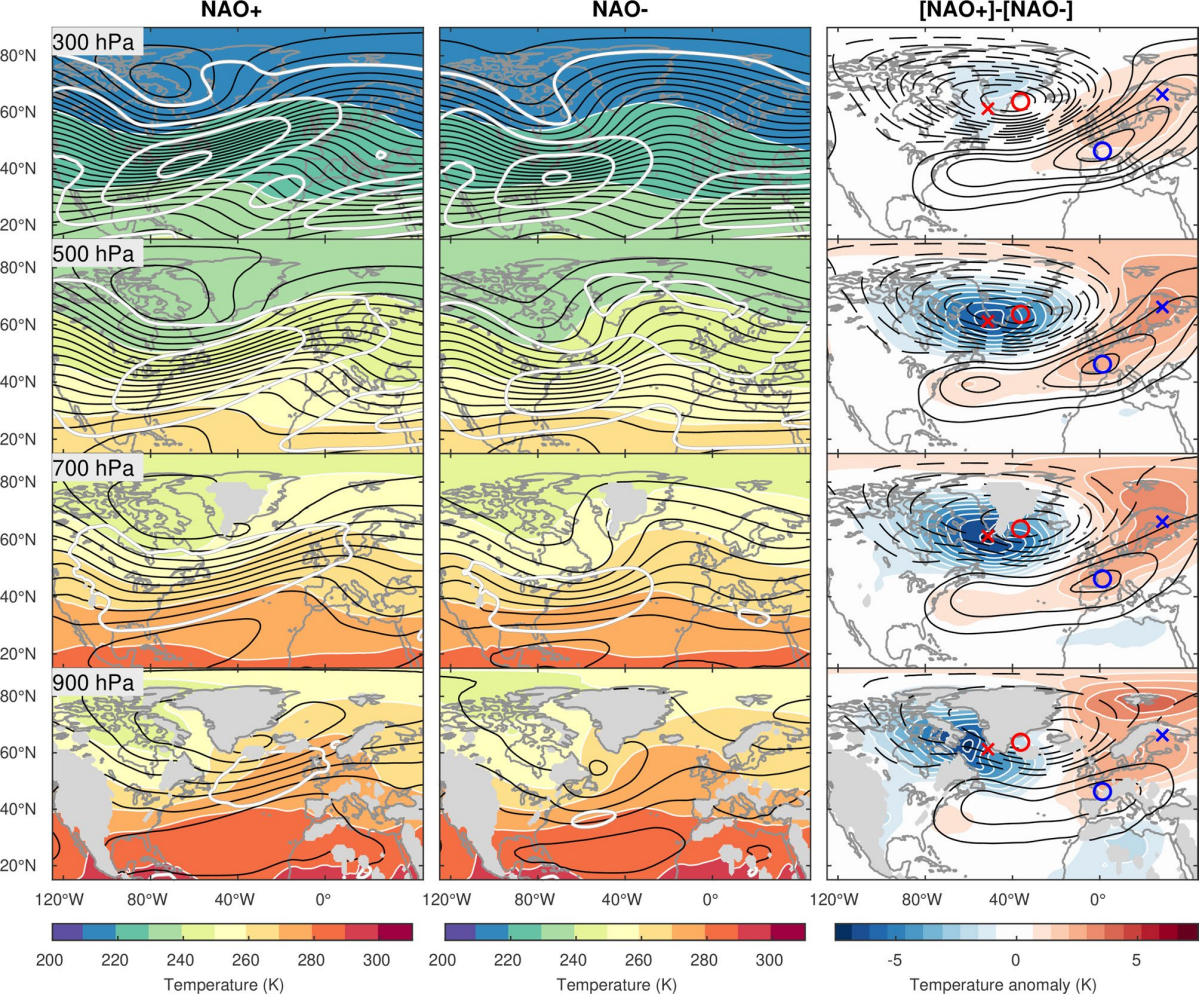
Wintertime structures of the NAO and the associated circulation and thermal anomalies. (Left and middle columns) Composite fields for the positive (NAO+) and negative (NAO−) phases of the NAO in winter (December–January–February). Geopotential height decreases poleward, as contoured in black with an increment of 50 m. Temperature is shaded at 10 K intervals. Isotachs are contoured in white with an increment of 10 m s^−1^. (Right column) Composite difference between the positive and negative NAO phases to typify the NAO+ anomalies (as NAO+ minus NAO−). Geopotential height anomalies are contoured in black for every 25 m with solid (dashed) lines for positive (negative) anomalies. Temperature anomalies are shaded at 0.75 K intervals. All composites are shown separately for pressure levels from 900 hPa (bottom) to 300 hPa (top; near the tropopause). The centers of cyclonic and cold anomalies at 500 hPa are indicated with red circles and crosses, respectively, at all levels. Similarly, the 500-hPa centers of anticyclonic and warm anomalies are shown with blue circles and crosses, respectively.

**Figure 2:**
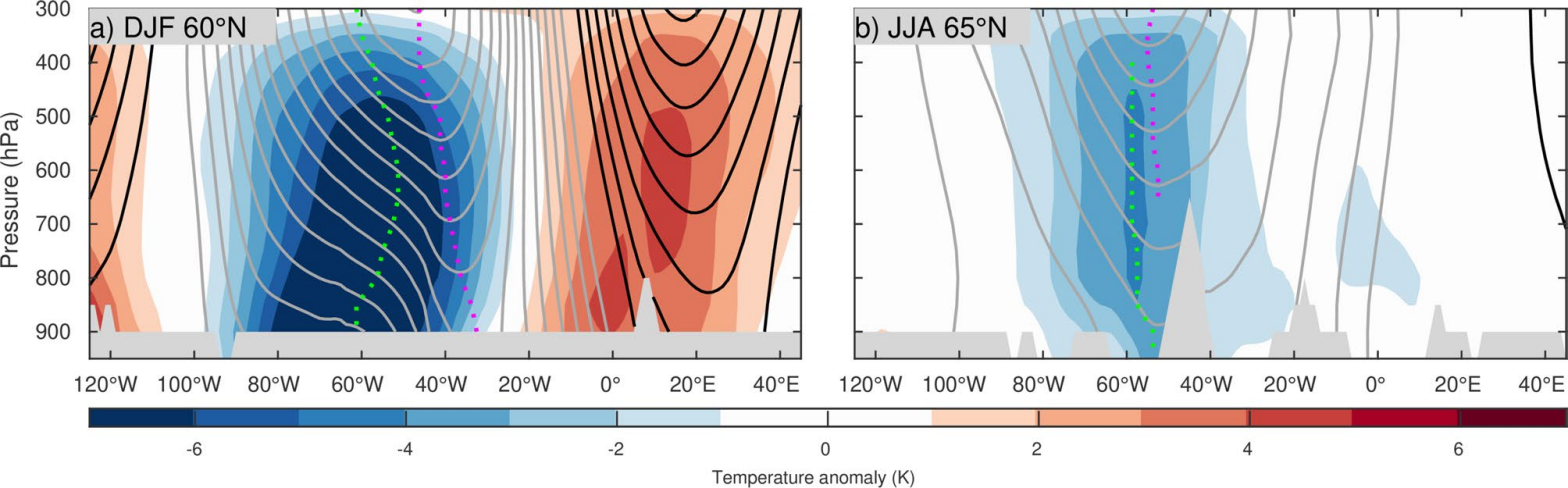
Zonal structure of NAO-associated circulation and thermal anomalies. Composite differences between the positive and negative phases to represent NAO+ anomalies (as NAO+ minus NAO−) at 60° N for winter (DJF) (**a**) and at 65° N for summer (JJA) (**b**), where the geopotential height and temperature anomaly minima are identified for each season. Geopotential height anomalies are contoured every 20 m using black and grey for positive and negative values, respectively. Temperature anomalies are shaded with 1 K intervals. Height and temperature anomaly minima at each pressure level are marked with dashed magenta and green lines, respectively.

Here, using atmospheric reanalysis data, we first investigate the typical structure of the circulation and temperature anomalies associated with the NAO in winter, when the NAO signature is most prominent (Fig. 1). Salient differences in the circulation between the positive and negative phases of the NAO are seen in the composited total fields: the positive phase (NAO+) is characterized by a well-defined eddy-driven jetstream that is deflected strongly poleward over the North Atlantic and extends farther northeastward in comparison to the negative phase (NAO−)^4,34,35^. Differences in the circulation and temperature anomalies between the NAO+ and NAO− composites (see “Methods”) highlight the typical NAO anomalies, which, by definition, represent the mature stage, or the peak, of the positive NAO phase. These anomalies are characterized by a meridional dipole in geopotential height anomalies corresponding to the meridional shift of the North Atlantic eddy-driven jetstream. The dipolar anomalies are largely equivalent barotropic with a high degree of vertical coherence. A careful inspection, however, reveals a subtle westward tilt of the northernmost center of the height anomalies with altitude in addition to an eastward tilt of the associated cold anomalies (see also Fig. 2a). Such structure is reminiscent, albeit weaker, of the vertically-tilting baroclinic structure characteristic of developing extratropical cyclones. Moreover, the height and temperature anomalies near the surface (at 900 hPa) are almost in quadrature, suggestive of lower-tropospheric heat transport by the NAO. Though not as obvious as the longitudinal tilts in Fig. 1, an equatorward tilt is also recognizable for the negative geopotential height anomalies over Greenland in meridional sections (Supplementary Fig. S1). Figures 1 and 2a thus suggest a potential involvement of baroclinic processes in the NAO dynamics.

**Figure 3:**
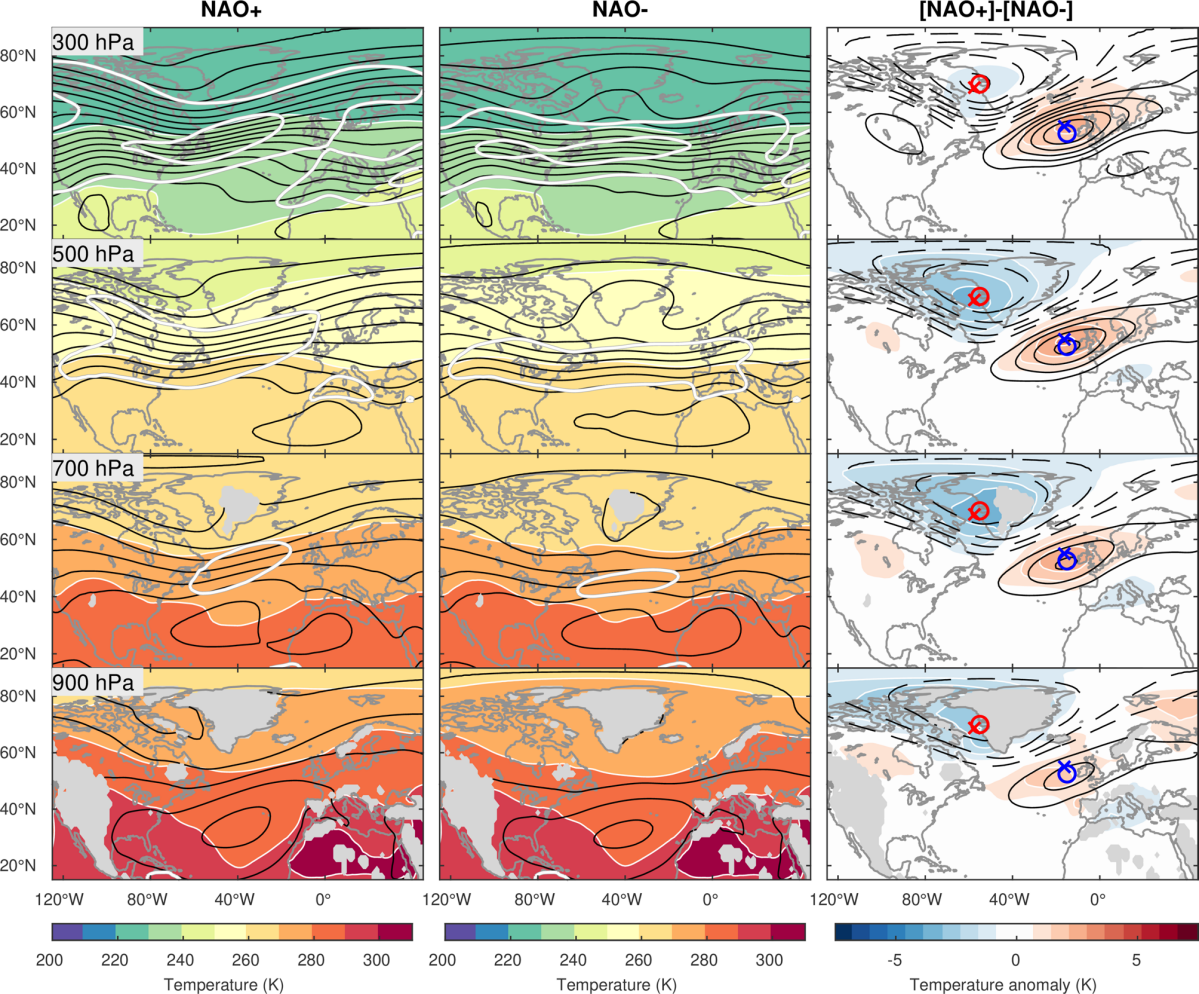
Summertime structure of NAO-associated circulation and thermal anomalies. Same as in Fig. 1 but for summertime (June–July–August).

In contrast to the wintertime NAO, differences in circulation between the summertime NAO+ and NAO− are rather weak (Figs. 2b, 3), while the eddy-driven jetstream remains shifted poleward during the NAO+ phase as in winter. Compared with their wintertime counterpart, the dipolar height anomalies exhibit greater NW-SE tilt, with the northern anomaly center being shifted westward towards Northeastern Canada^36^. The structure of the summertime NAO circulation and thermal anomalies is more equivalent barotropic with a higher degree of vertical coherence throughout the troposphere and in-phase height and temperature anomalies in the lower troposphere, implying a lesser involvement of baroclinic processes in comparison to winter.

### Processes energizing the NAO anomalies

**Figure 4:**
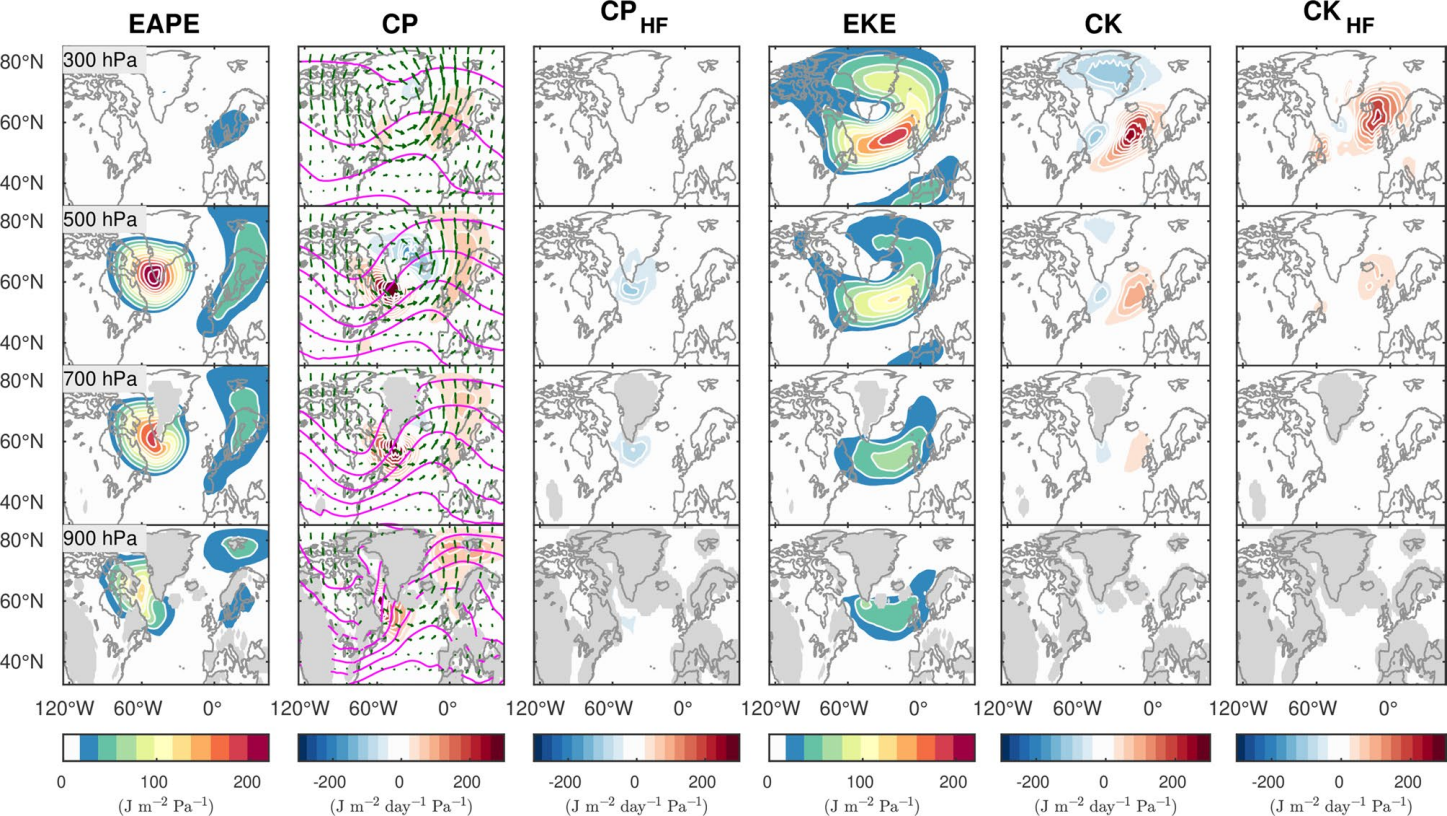
Energy, its conversion, and feedback associated with the wintertime NAO circulation anomalies. Eddy available potential energy (EAPE, 1st column, J m^−2^ Pa^−1^) and eddy kinetic energy (EKE, 4th column, J m^−2^ Pa^−1^) associated with the NAO anomalies in winter, and contributions from baroclinic energy conversion (CP, 2nd column, J m^−2^ Pa^−1^ day^−1^) and barotropic energy conversion (CK, 5th column, J m^−2^ Pa^−1^ day^−1^) to the maintenance of the NAO energy. Climatological-mean temperature, which is decreasing poleward, is superimposed on CP with magenta contours at 5 K intervals. Wind anomalies associated with the NAO are shown with green arrows. A distance of 1° corresponds to a wind speed of 2 m s^−1^. Feedback (J m^−2^ Pa^−1^ day^−1^) from high-frequency transient eddies to the maintenance of EAPE (CP_HF_, 3rd column) and EKE (CK_HF_, 6th column) are also shown. Energetics are shown separately for pressure levels from 900 hPa (bottom) to 300 hPa (top).

To assess the relative importance of various processes in the maintenance of the NAO anomalies, we first illustrate the three-dimensional distributions of the NAO’s energy and local contributions from forcing terms of the NAO’s energetics in winter (Fig. 4). To evaluate the energetics, the NAO anomalies are defined as a difference between the NAO+ and NAO− composites shown in Fig. 1. By definition, the energy associated with these NAO anomalies is directly related to their squared amplitude (c.f. “Methods”).

The eddy available potential energy (EAPE) associated with the NAO, which is proportional to the squared temperature anomalies shown in Fig. 1, is maximized around the southern tip of Greenland, while its near-surface maximum is shifted westward over the Labrador Sea (Fig. 4, 1st column). EAPE intensifies from the near-surface up to 500 hPa under the decreasing stability parameter (Supplementary Fig. S2), while weakening above mainly because of diminishing temperature anomalies.

Baroclinic energy conversion (CP), the conversion of available potential energy (APE) from the mean flow to the NAO-associated EAPE, is strongly positive in the mid- and lower troposphere near the southern tip of Greenland and thus well aligned with the NAO’s EAPE maximum (Fig. 4, 2nd column). Over the Euro-Atlantic sector, positive CP dominates over negative CP, yielding a net positive CP to the NAO’s EAPE. The positive CP results from the NAO-associated low-level anomalous winds that blow across the climatological temperature gradient associated with the climatological eddy-driven westerly jetstream over the subpolar Euro-Atlantic sector, thereby acting to relax it. The cyclonic anomaly associated with NAO+ is a manifestation of the intensified Icelandic Low, a semi-permanent surface low-pressure system, and thus strengthens both the northwesterlies from cooler eastern Canada and Greenland toward the warmer Atlantic and the southwesterlies over northern Europe toward the cooler Arctic (Fig. 1). The resultant enhancement of the cold and warm advection around the Icelandic Low (Supplementary Fig. S3) acts to reinforce the cool and warm anomalies existing over Canada/Greenland and Europe, respectively. During NAO−, by contrast, the cold and warm advection is weakened by the anticyclonic anomalies, but acts again to reinforce the warm and cool anomalies existing over Canada/Greenland and Europe, respectively. Both longitudinal and meridional heat fluxes contribute to positive CP (Supplementary Fig. S4), indicating that both the westward and southward vertical tilts of the NAO circulation anomalies are important for extracting energy from both the meridional and zonal background temperature gradients associated with the climatologically deflected westerly jetstream due to stationary waves. The processes contributing to CP are summarized schematically in Supplementary Fig. S5.

Eddy kinetic energy (EKE) associated with the NAO circulation anomalies, which is proportional to the squared anomalous wind velocity, is largest in the upper troposphere (Fig. 4, 4th column). The maximum EKE is found along the midlatitude node of the dipolar height anomalies shown in Fig. 1, where the geostrophic wind variability is strongest. Secondary EKE peaks are observed in the subtropics and Arctic. The EKE is sustained around its midlatitude maximum located in the joint exits of the stormtrack and eddy-driven westerly jetstream over the Northeastern Atlantic by both barotropic feedback forcing from migratory disturbances (CK_HF;_ Fig. 4, 6th column) and barotropic energy conversion (CK) from the mean jetstream (Fig. 4, 5th column). These conversions act to enhance EKE but are partially dissipated through CK slightly upstream and at high latitudes.

In contrast to CP, the contribution of high-frequency transient eddies to the maintenance of EAPE (CP_HF_) is small and negative, consistent with down-gradient heat fluxes of high-frequency baroclinic eddies that acts to damp the low-frequency thermal anomalies over the sector^37,38^. Nevertheless, high-frequency eddies contribute to the reinforcement of NAO’s EKE (CK_HF_) in the upper troposphere. This is consistent with the driving and maintenance role of migratory eddies^22,23^, which is strongest downstream of the EKE maximum.

**Figure 5:**
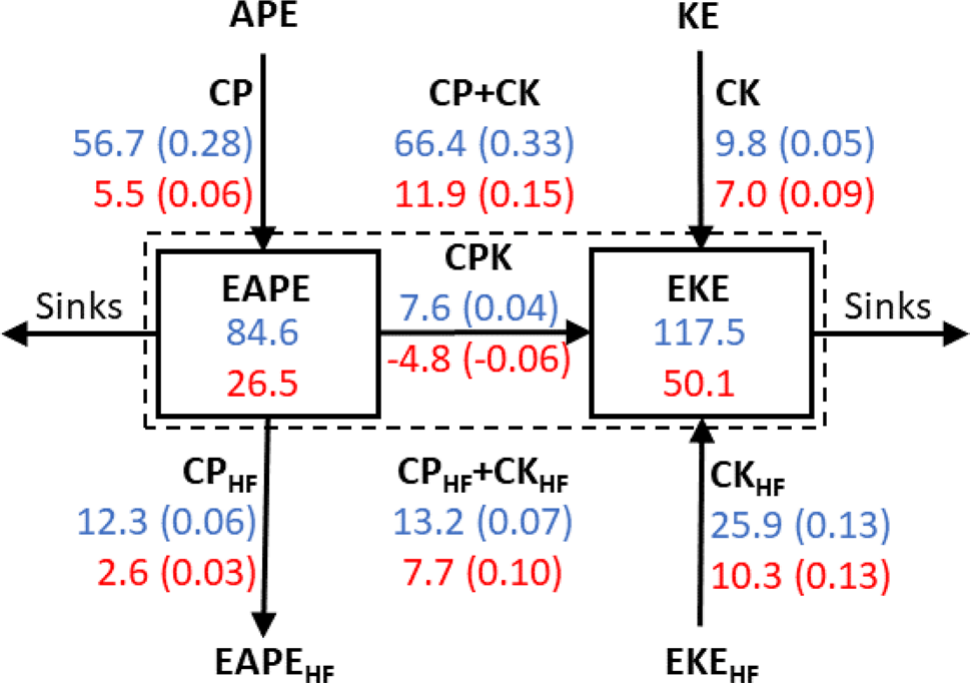
Contrasting wintertime and summertime energetics. Schematic depiction of the energetics of the NAO, after integrated over the Northern Hemisphere and vertically from the surface to 100 hPa (~ 15 km in altitude), separately for winter (blue, DJF) and summer (red, JJA). Numbers in EAPE and EKE boxes represent energy storage in units of 10^17^ J. The total energy storage of the NAO (EAPE + EKE) is indicated with a dashed box. Energy conversion (CP and CK), feedback CP_HF_ and CK_HF_), and transfer (CPK)  are represented with arrows, whose direction indicates whether they are sources or sinks and are indicated with units of 10^17^ J day^−1^. Numbers in parentheses indicate the efficiency of energy conversion, feedback, and transfer (energy sources are divided by the total energy of NAO (EAPE + EKE; box with dashed lines) with units of day^−1^. APE and KE denote the available potential energy and the kinetic energy of the background flow, whereas EAPE_HF_ and EKE_HF_ denote the available potential energy and kinetic energy of high-frequency eddies. A negative value indicates that a particular conversion, feedback, or transfer is in the direction opposite to the direction indicated with the corresponding arrow.

The magnitudes of the energy conversion terms, CP and CK, are, by definition, proportional to the squared amplitude of the NAO thermal or wind anomalies that are strongly dependent on the seasonal cycle. For a fair comparison of the energetics among different seasons it is therefore necessary to evaluate the *efficiency* that is defined here as the energy conversion divided by the total energy (EAPE + EKE) of the NAO integrated hemispherically and vertically. The inverse of this efficiency corresponds to the time scale^39^ over which a certain process alone could fully replenish the total energy, provided that the NAO structure does not change throughout its growth. Note that the energy sources evaluated here are associated with the mature phase of the NAO when its energetics are largely equilibrated. Most of the energy sources thus serve to maintain the NAO-associated circulation anomalies against dissipative processes. In winter, CP clearly emerges as the dominant energy source with an efficiency of 0.28 day^−1^ (Fig. 5), confirming the important role of baroclinic processes in low-frequency variability over the sector^40,41^. With such a high efficiency, CP could fully replenish the total NAO energy within only 4 days, equally contributed by both longitudinal and meridional heat fluxes (not shown). In contrast to CP, CK’s efficiency is of only 0.05 day^−1^, which indicates that the transfer of kinetic energy from the mean jetstream to the NAO plays only a minor role in energizing the wintertime NAO anomalies.

CP is also substantially more efficient than the *net* feedback forcing from high-frequency eddies (CP_HF_ + CK_HF_), as they overall remove APE from the NAO through down-gradient heat fluxes (CP_HF_, − 0.06 day^−1^) but enhance EKE (CK_HF_, 0.13 day^−1^). In fact, the net contribution from the eddies (CP_HF_ + CK_HF_) is positive but its time scale is about 2 weeks. Our analysis therefore suggests that the role of migratory eddies may be overestimated if one focuses only on their barotropic feedback (CK_HF_) in the upper troposphere, where it is largest (see CK_HF_, Fig. 4), while neglecting the role of baroclinic processes in energizing the NAO.

The summertime NAO energetics is marked by a five-fold decrease of CP (0.06 day^−1^) compared to its wintertime counterpart (0.28 day^−1^), due to the reduced vertical tilt of the summertime NAO anomalies under the weaker background thermal gradient, as discussed earlier. Modest CP efficiencies in spring (0.19) and fall (0.24) reflect the transition from the more barotropic structures of the NAO and background flow in the warm season to their more baroclinic structures in the cold season. In contrast to CP, the efficiencies of CK, CP_HF_, and CK_HF_ show weaker seasonality. The sign of the transfer from EAPE to EKE (CPK) is flipped between winter and summer, reflecting the dominance of the baroclinic processes in winter compared to the barotropic processes in summer for maintaining the large-scale thermal and circulation anomalies of the NAO against dissipative processes.

## Discussion

The energetics shown above elucidate why the NAO is such a prominent mode of variability with persistent thermal and circulation anomalies. Specifically, the NAO in the cold season is a preferred dynamical mode over the Euro-Atlantic sector that can efficiently maintain the associated thermal anomalies against dissipative processes through anomalous thermal advection across the climatologically-deflecting westerly jetstream. The wintertime enhancement of the vertical tilt of the NAO anomalies, which allows an effective conversion of potential energy from the mean flow, is likely a contributing factor to seasonal changes in the nature of the NAO-associated anomalies, such as their amplitude and persistence. In addition to more prominent temperature and wind anomalies in winter, we also find, by computing its *e*-folding time, that the NAO index is more persistent in winter (5.7 days) than in summer (4.6 days), which is consistent with a more efficient energy gain through the baroclinic conversion in winter.

The vertically-tilting NAO anomalies can be understood as a modulation of the climatological stationary waves, since the NAO+ and NAO− correspond to the strengthening and weakening of the Icelandic Low, respectively. These waves forced by land–sea thermal contrasts and large-scale orography are energized climatologically, as a consequence of asymmetries in the flow, by baroclinic energy conversion resulting from poleward heat transport^42^. The heat fluxes associated with the NAO are thus consistent with the modulated stationary waves over the Euro-Atlantic sector. From this viewpoint, one may consider the interactions between transient eddies and the climatological stationary waves as the initial forcing, and the conversion of EAPE resulting from the circulation anomalies as a subsequent feedback. The NAO’s North Pacific counterpart, the North Pacific Oscillation or the Western Pacific pattern^1^, has been shown to be maintained primarily through baroclinic energy conversion via heat transport^43^, as we found for the NAO.

We stress that our findings do not minimize the importance of migratory transient eddies in *driving* the NAO^22^. Strong evidence indicates that wave breaking is the key process for the NAO phase transitions^19,28–30,32,35^. Occurring around the baroclinic westerly jetstream, the wave-breaking process itself, and the vertical structure of the breaking waves, may play an important role in setting the baroclinic features of the NAO. Future work therefore needs to address the efficiency of the various processes discussed here not only for the peak, or mature stage, of the NAO phases, as has been examined here, but also throughout the NAO life cycle, and how transient eddies, through their development and breaking, impart their vertical structures onto the NAO variability.

We have also analyzed the positive and negative phases of the NAO separately and found that both are maintained primarily by baroclinic energy conversion with similar efficiencies (not shown). It is not guaranteed, however, that every NAO event is energized as efficiently by this mechanism due to diversity in the structures of weather events projecting on the NAO^44^. Future work should explore how this diversity affects the role of baroclinic energy conversion in the amplification and persistence of individual events.

Our findings have revealed a fundamental mechanism of the NAO dynamics and are thus expected to contribute to the improvement of subseasonal to seasonal prediction of the Euro-Atlantic climate and the representation of the NAO variability in climate models by providing a benchmark to assess their ability to adequately reproduce the three-dimensional structure of the NAO. Given the important role of baroclinic processes in energizing the North-Atlantic circulation anomalies as evidenced by this study, it appears relevant to investigate how the changing climatic conditions will influence the vertical tilt of the NAO anomalies and the background energy available for the baroclinic energy conversion to the NAO. There is evidence from observations that the North Atlantic eddy-driven jetstream has been gradually migrating poleward in summer and fall^45^ and is predicted to migrate farther poleward according to climate model projections^46–48^. Changes in the large-scale baroclinicity and thus in the APE are also projected to occur^48^ for all seasons. These future changes in the background state may influence how the NAO will derive its energy from the background flow and how it will produce weather variability over the Euro-Atlantic sector through changes in its amplitude and persistence.

## Methods

### Data

This study uses atmospheric data from the JRA-55 global reanalysis dataset^49^ from 1958 to 2016. Variables analyzed include the three-dimensional wind field ($u,v,\omega$), temperature (T), geopotential height (*Z*), and parameterized heating rate (*Q*). All variables are available every 6 h at a spatial resolution of 1.25° in latitude and longitude. This study uses 18 evenly spaced pressure levels from 1,000 to 100 hPa.

### NAO index

The NAO index is defined as the projection of daily 500 hPa height anomalies, defined as departures from a smoothed seasonal cycle, onto the NAO pattern. The seasonal cycle is obtained by averaging daily values over all years and retaining the lowest four Fourier frequencies. The NAO pattern (Supplementary Fig. S6) is obtained as the first empirical orthogonal function (EOF) of monthly-mean 500 hPa height anomalies evaluated independently for the months of December–January–February (DJF), June–July–August (JJA), March–April–May (MAM) and September–October–November (SON). The EOF is performed for the Euro-Atlantic sector (20° N–90° N, 90° W–30° E). The NAO index thus obtained is similar to the one provided by the U.S. National Oceanic and Atmospheric Administration (NOAA), publicly available at https://www.cpc.ncep.noaa.gov/products/precip/CWlink/pna/nao.shtml. The correlation between the two indices is 0.8 in DJF and 0.87 in JJA. Our results are not sensitive to the choice of index.

### Defining NAO-related circulation anomalies

The NAO index varies on a wide range of time scales from days to decades and even longer. The typical life cycle of the NAO is, however, of about 2 weeks^22,50^. To focus our analysis on this time scale, we first apply a 10–60 day band-pass Butterworth filter onto the NAO index. The resulting time series and its amplitude spectrum are shown in Supplementary Fig. S7. From inspection of the amplitude spectrum, it is clear that the filter used is inclusive of the typical lifetime of NAO. Circulation anomalies associated with this time scale of NAO variability are then identified through a composite method. Whenever the filtered NAO index rises (falls) above (below) 0.5 (−0.5) standard deviation, the dates in question are selected for the NAO+ (NAO−) composite. Most of our analysis is carried out for the typical NAO anomalies obtained by subtracting the NAO− composite from the NAO+ composite to feature the latter, but nonlinearities between the two phases are also briefly investigated. The NAO anomalies, as defined here, thus represent the mature stage, or the peak, of the NAO+.

### Energetics of NAO

Transfers of energy between the climatological-mean state and the NAO anomalies are evaluated by using atmospheric energetics formulated for the time domain^51^. Transfer of kinetic energy from the mean flow to NAO-associated wind anomalies is called barotropic energy conversion (CK) and evaluated with:

1 $$CK=\frac{{v}^{{^{\prime}}2}-{u}^{{^{\prime}}2}}{2}\left(\frac{\partial \overline{u}}{\partial x}-\frac{\partial \overline{v}}{\partial y}\right)-{u}^{{\prime}}{v}^{{\prime}}\left(\frac{\partial \overline{u}}{\partial y}+\frac{\partial \overline{v}}{\partial x}\right),$$

where overbars denote the time mean over a season (DJF for winter, MAM for spring, JJA for summer, and SON for fall) for 1979–2016, and primes denote NAO-related anomalies as defined above. Positive values denote transfers of kinetic energy from the mean flow to the eddy kinetic energy (EKE) of the NAO, which is defined as:

2 $$EKE=\frac{{u}^{{^{\prime}}2}+{v}^{{^{\prime}}2}}{2}.$$

Similarly, conversion of available potential energy (APE) from the mean flow to the eddy available potential energy (EAPE) of NAO, called the baroclinic energy conversion (CP), is evaluated with

3 $$CP=-{\gamma }^{-1}\left({u}^{{\prime}}{T}^{{^{\prime}}}\frac{\partial \overline{T}}{\partial x}+{v}^{{\prime}}{T}^{{^{\prime}}}\frac{\partial \overline{T}}{\partial y}\right),$$

where the stability parameter (*γ*) is defined as:

4 $$\gamma =\frac{p}{R}\left(\frac{R\widehat{\overline{T}}}{{C}_{p}p}-\frac{d\widehat{\overline{T}}}{dp}\right),$$

where *R* is the gas constant for dry air (287 J K^−1^ kg^−1^), *C*_*p*_ is the specific heat at constant pressure (1,004 J K^−1^ kg^−1^), and the hat operator denotes an area average over the Northern Hemisphere. The terms related to the zonal and meridional heat fluxes in (3) are hereafter referred to as the zonal (CPx) and meridional (CPy) components, respectively. The NAO-associated EAPE is expressed as

5 $$EAPE={\gamma }^{-1}\frac{{T }^{{^{\prime}}2}}{2}.$$

Transfer from EAPE to EKE is evaluated as

6 $$CPK=-\frac{R{\omega }^{{\prime}}{T}^{{\prime}}}{p}.$$

In addition to the energy conversions from the mean flow, the baroclinic feedback of high-frequency (HF) eddies^43^ onto the NAO-associated EAPE is evaluated with

7 $$C{P}_{HF}=-{\gamma }^{-1}T{^{\prime}}\left(\frac{\partial {({u}^{\prime\prime}{T}^{\prime\prime})}^{{\prime}}}{\partial x}+\frac{\partial {({v}^{\prime\prime}{T}^{\prime\prime})}^{{\prime}}}{\partial y}\right)$$

and the barotropic feedback^43^ of HF eddies onto the NAO-associated EKE is evaluated with:

8 $$CK_{{HF}} = - u^{\prime } \left( {\frac{{\partial \left( {u^{{\prime \prime }} u^{{\prime \prime }} } \right)^{\prime } }}{{\partial x}} + \frac{{\partial \left( {u^{{\prime \prime }} v^{{\prime \prime }} } \right)^{\prime } }}{{\partial y}}} \right) - v^{\prime } \left( {\frac{{\partial \left( {u^{{\prime \prime }} v^{{\prime \prime }} } \right)^{\prime } }}{{\partial x}} + \frac{{\partial \left( {v^{{\prime \prime }} v^{{\prime \prime }} } \right)^{\prime } }}{{\partial y}}} \right).$$

In (7) and (8), double primes denote the high-frequency component of the circulation obtained with a 10-day high-pass Butterworth filter, and primes denote a composite difference of these high-frequency covariance fields between the two phases of NAO.

In addition to the resolved components of the energetics, the effect of diabatic heating and heating due to vertical diffusion, which are parameterized in JRA-55, are evaluated as.

9 $$CQ={\gamma }^{-1}\frac{{Q}^{{\prime}}{T}^{{\prime}}}{{C}_{p}},$$

where *Q* is the diabatic heating rate.

Note that the energetics illustrated in Fig. 5 do not form a closed budget. In winter, the sum of the EAPE sources and sinks, including the diabatic forcing (CQ; efficiency of − 0.05 day^−1^), results in an imbalance of 0.13 day^−1^ in the EAPE budget. This imbalance can arise from possible inaccuracies in* Q* components in JRA-55, including convective processes, radiative heating and surface heat fluxes. The corresponding imbalance for EKE is larger, at 0.22 day^−1^. This imbalance could be attributed to the terms that were excluded from our analysis^51–53^, including the vertical redistribution of wind momentum by gravity waves and frictional dissipation. In comparison to winter, the summertime imbalance is greatly reduced for the EAPE (0.04 day^−1^) but similar for the EKE (0.17 day^−1^). These imbalances may also be attributable partly to the slow evolution of the NAO structure, which is neglected in our energetics. A complete assessment of the energy budget is, however, beyond the scope of this study.

### NAO time scale

We define the time scale of NAO variability as the “*e*-folding time” of the NAO index, which is the lag at which the autocorrelation function of the index decreases to 1/*e*. Interannual variability has been removed by subtracting the seasonal mean from each calendar day before calculating the lag-correlation. We find that the *e*-folding time is reduced substantially by isolating the intraseasonal variability of the NAO, compared to using the raw NAO index (5.7 days versus 9.6 days in DJF). Similar values are obtained by assessing the *e*-folding time separately for each year. As illustrated in Supplementary Fig. S8, the interannual NAO variability enhances the lag-correlation substantially, even at short lags, through artificially inflating the NAO’s persistence. Similarly, interannual variability can also affect the assessment of eddy feedback by lagged correlation^54^. This result suggests that the *e*-folding time scale reported previously^50^, which is similar to the one computed here from the raw NAO index, may overestimate the typical time scale of the NAO events. A similar reduction of the *e*-folding time is also observed in JJA (4.6 days versus 6.4 days).

### Sensitivity analysis

We briefly consider asymmetries between the two phases of the NAO by evaluating the structure of the NAO and its energetics separately for NAO+ and NAO−. The structure is essentially similar between NAO+ and NAO− (not shown), except that the negative phase shows slightly stronger anomalies, consistent with the negative skewness of NAO^35^. Our analysis suggests that differences in the energetics between the two phases of NAO are overall small and not significant (not shown). The sensitivity of our results to the time scale used to define the NAO anomalies (not shown) is also investigated, which reveals the enhancement of the efficiency of baroclinic energy conversion for the 10–60 day period in our main analysis (0.28 day^−1^) in comparison to the same analysis carried for monthly averages (0.18 day^−1^) or the 60 day–1 year frequency band (0.21 day^−1^). We also briefly investigated the efficiency of baroclinic energy conversion during the development (NAO > 0.5, *d*NAO*/dt* > 0 or NAO < –0.5, *d*NAO*/dt* < 0) and decay (NAO > 0.5, *d*NAO/*dt* < 0 or NAO < –0.5, *d*NAO/*dt* > 0) of NAO phases and observed similarly large efficiencies in all cases (not shown).

One may question whether the high-frequency eddy feedback forcing, which is often assessed by lag-correlation^23,55^, is assessed adequately in the composite analysis. By focusing here on subseasonal NAO variability, an adequate time scale separation is ensured, providing high-frequency eddies sufficient time for reacting to the changing large-scale circulation and thereby exerting feedback forcing on the NAO anomalies. Slower time scales, such as monthly or interannual NAO variability, provide even more time for the feedback to become more effective, but we find a tendency for the high-frequency eddy forcing (CP_HF_ + CK_HF_) to be rather insensitive to the time scale used to define the NAO.

## Supplementary information

Supplementary file1
