# Supplementary material for: Importance of a vertically tilting structure for energizing the North Atlantic Oscillation
Source: Sci Rep. 2020 Jul 29;10:12671. doi: 10.1038/s41598-020-69551-5 (PMC7391741; doi:10.1038/s41598-020-69551-5)
Supplement: Supplementary file 1 — Supplementary file1 [file 41598_2020_69551_MOESM1_ESM.docx]

Supplementary Information: Importance of a vertically tilting structure for energizing the North Atlantic Oscillation

Patrick Martineau^1*^, Hisashi Nakamura^1,2^, Yu Kosaka^1^, and Ayako Yamamoto^2^

1 Research Center for Advanced Science and Technology, University of Tokyo, Tokyo, Japan

2 Japan Agency for Marine-Earth Science and Technology, Yokohama, Japan

*corresponding author: pmartineau@atmos.rcast.u-tokyo.ac.jp

**
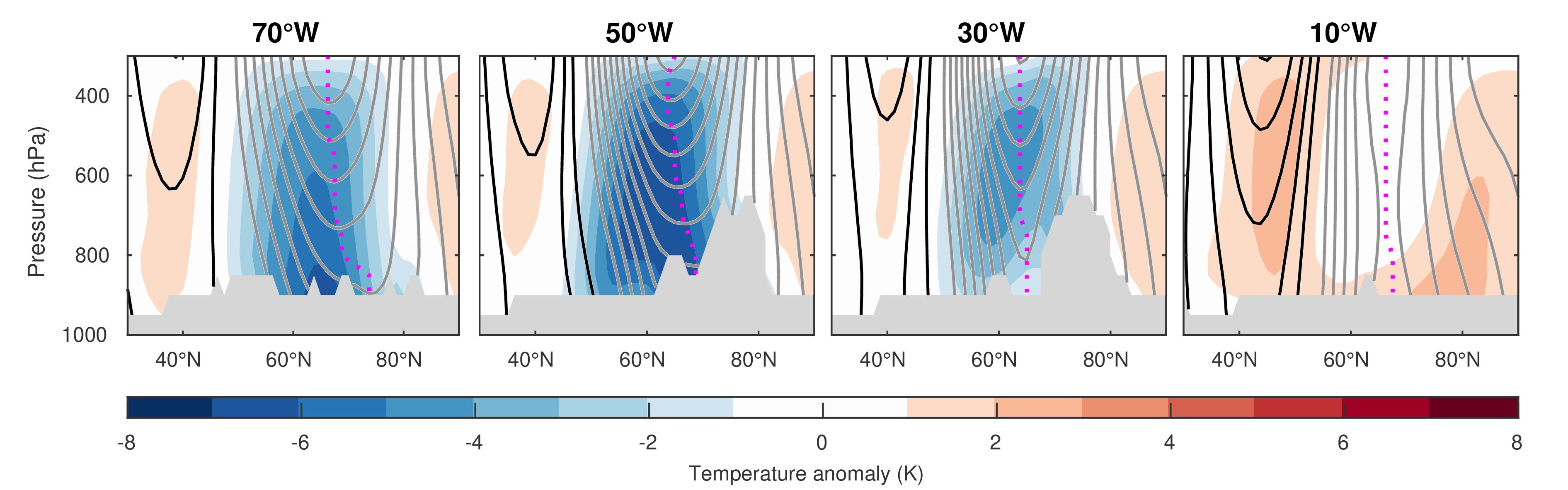
**

**Supplementary Figure 1: Meridional structure of NAO**

The NAO anomaly composites of temperature (shading) and geopotential height (contours) in the meridional sections shown for specific longitudes as indicated (rows). The difference between the composited NAO+ and NAO– is illustrated. Geopotential height anomalies are contoured every 25 m using black and grey for positive and negative values, respectively. Height anomaly minima at each pressure level are indicated with dashed magenta lines.

**
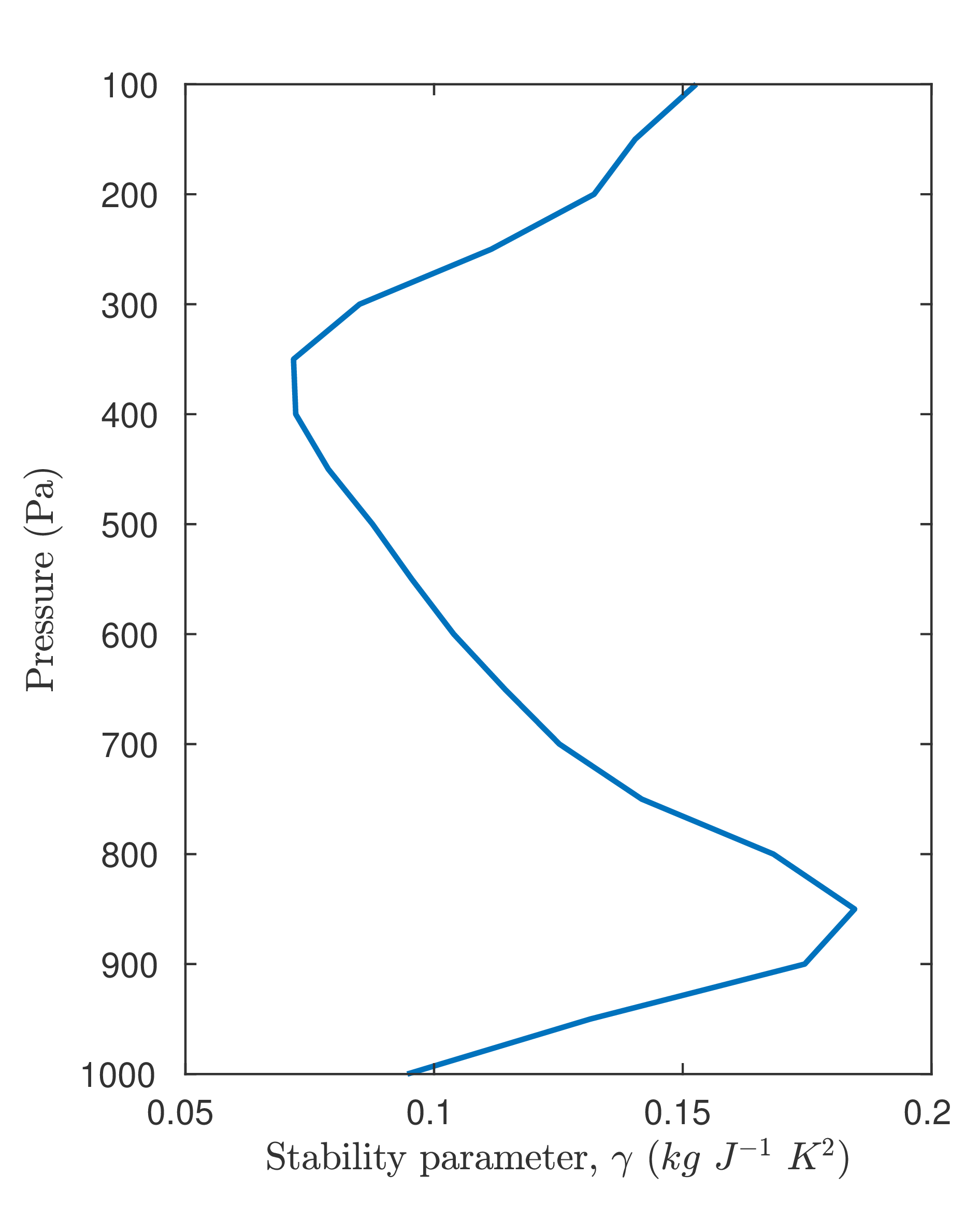
**

**Supplementary Figure 2: Wintertime climatology of the stability parameter**

The wintertime (December-January-February) climatology (1958-2016) of the stability parameter $\gamma$ (eq. (4)) is shown as a function of pressure levels.


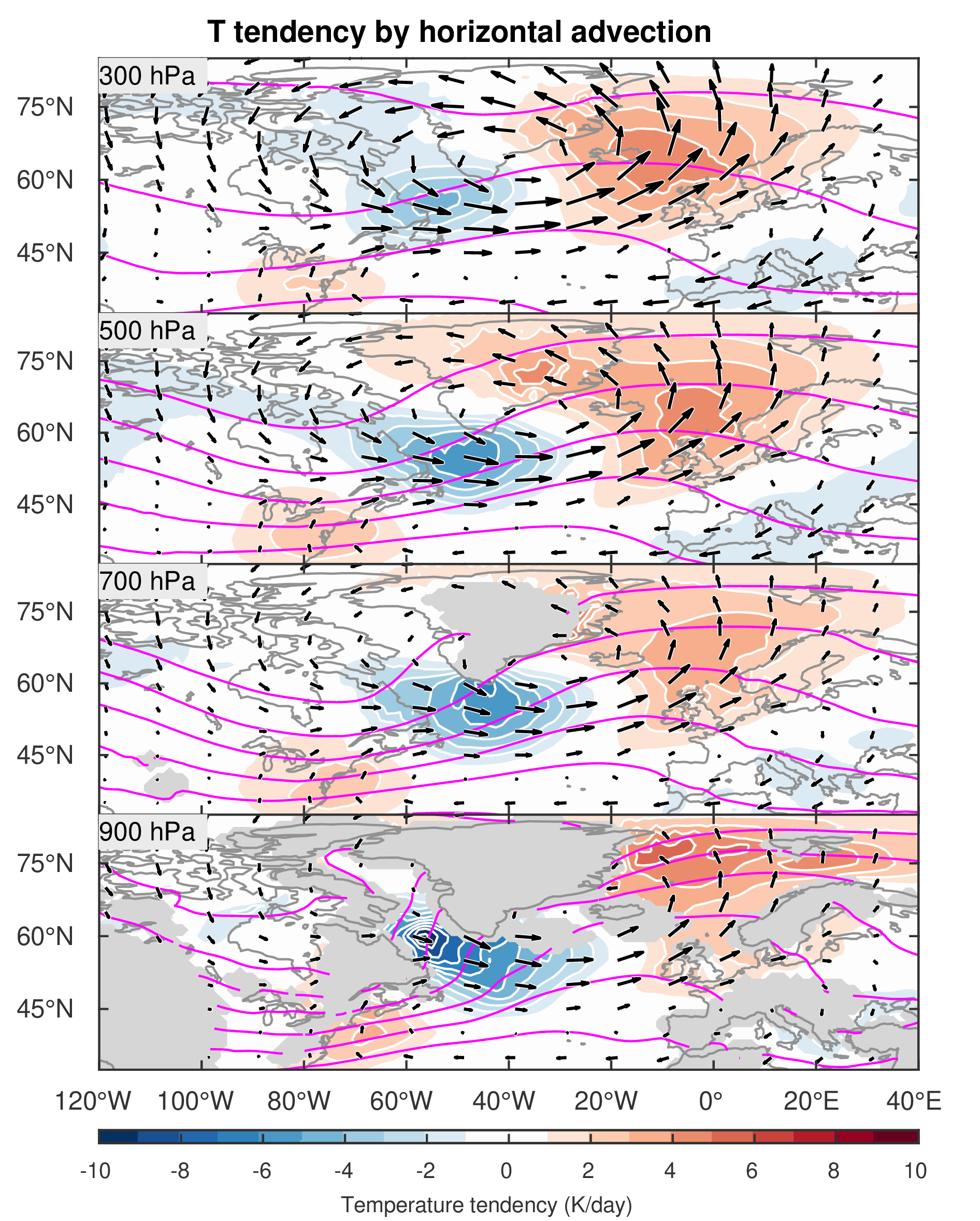


**Supplementary Figure 3: Anomalous temperature advection by NAO**

Anomalous advection (shadings, $-\boldsymbol{u}'\cdot\nabla\bar{T}$) of the climatological temperatures by NAO-associated wind anomalies is shown separately for individual pressure levels from 900 hPa (bottom) to 300 hPa (top; near the tropopause). The climatological-mean temperature ($\bar{T}$), which is decreasing poleward, is illustrated with magenta contours at 5 K intervals. NAO-associated wind anomalies ($\boldsymbol{u}'$) are shown with black arrows. A distance of 1° corresponds to a wind speed of 2 m s^-1^.


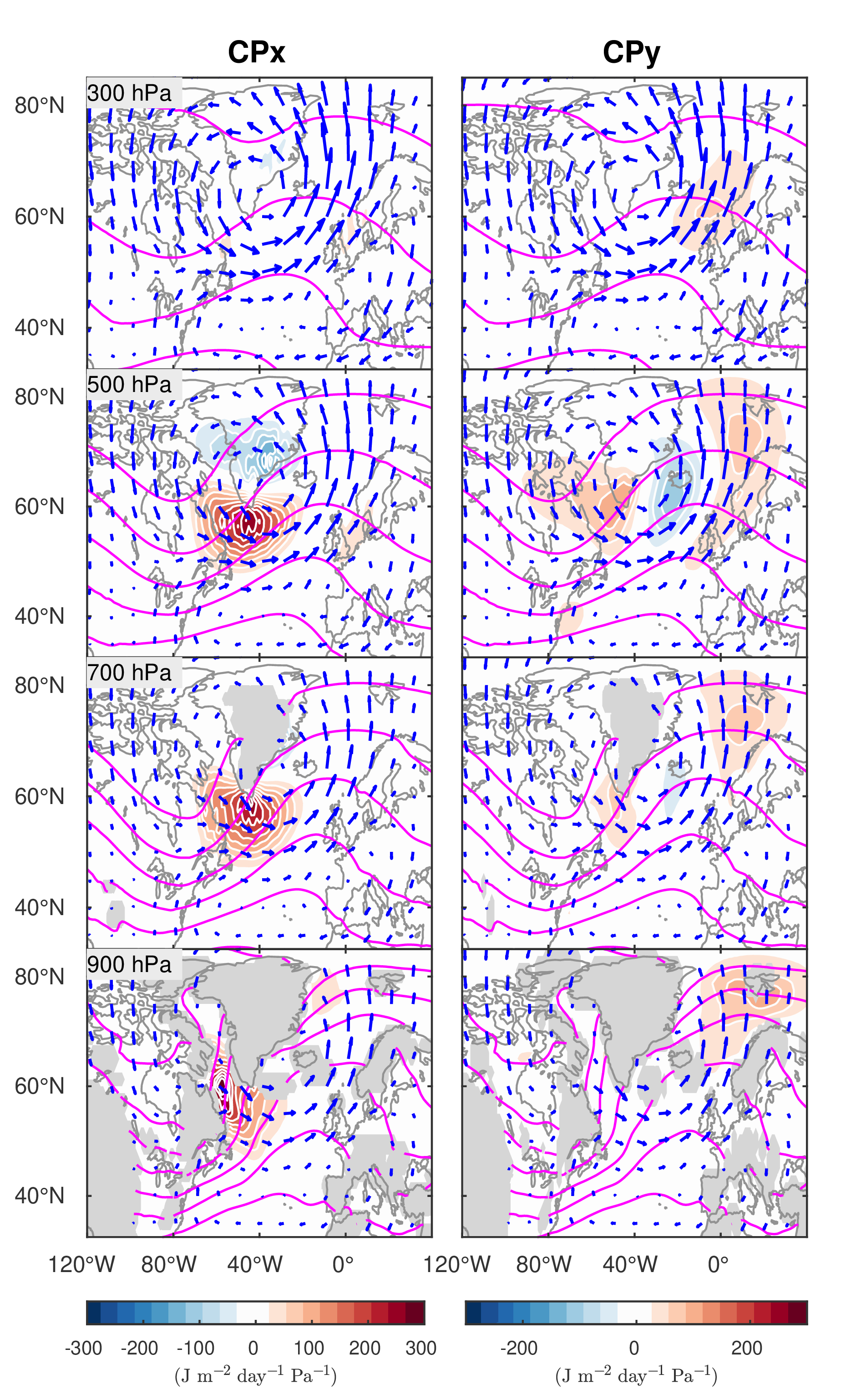


**Supplementary Figure 4: Decomposing baroclinic energy conversion into zonal and meridional components**

The zonal (CPx, J m^-2^ day^-1^ Pa^-1^) and meridional (CPy, J m^-2^ day^-1^ Pa^-1^) components of baroclinic energy conversion in Eq. (3) are shown separately. The climatological-mean temperature, which is decreasing poleward, is shown with magenta contours with an interval of 5 K. NAO-associated wind anomalies ($\boldsymbol{u'}$) are shown with blue arrows. A distance of 1° corresponds to a wind speed of 2 m s^-1^. Energetics are shown for specific pressure levels from 900 hPa (bottom) to 300 hPa (top).


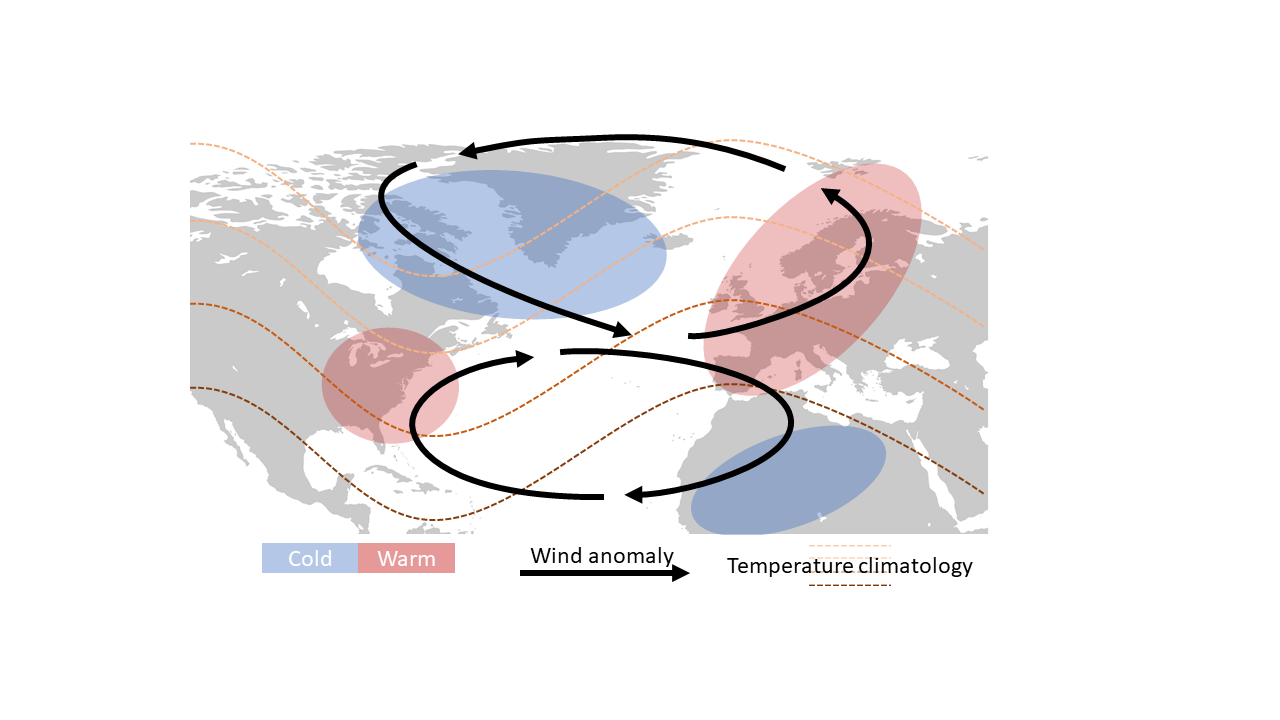


**Supplementary Figure 5**: **Illustrating the wintertime baroclinic energy conversion energizing the NAO**

A schematic illustrating the circulation anomalies in the lower troposphere associated with the positive phase of the wintertime NAO. The negative phase is the opposite, reflecting the mostly symmetric structures of the NAO phases. This diagram is representative of the lower troposphere, where the baroclinic nature of NAO is more evident. Cold and warm anomalies are illustrated with blue and red shades, respectively. Main circulation anomalies are shown with black arrows. Finally, the climatological-mean temperatures are illustrated with dashed lines that progressively darken toward the equator, corresponding to lowering temperature with latitude. Baroclinic energy conversion is achieved by downgradient heat fluxes resulting from NAO-related circulation anomalies. An alternative interpretation can be obtained by rewriting the baroclinic energy conversion in Eq. (3) as $\gamma^{-1}(-\boldsymbol{u}'\cdot\nabla\bar{T})T'$, which emphasizes that the conversion of available potential energy from the climatological-mean state to the NAO occurs when temperature anomalies ($T'$) are reinforced by anomalous thermal advection through the NAO-associated anomalous circulation acting on the background temperature gradient ($-\boldsymbol{u}'\cdot\nabla\bar{T}$). This process reinforces temperature anomalies over most of the Atlantic sector, most importantly over Greenland, where cold northwesterly advection reinforces the cold anomalies.

**
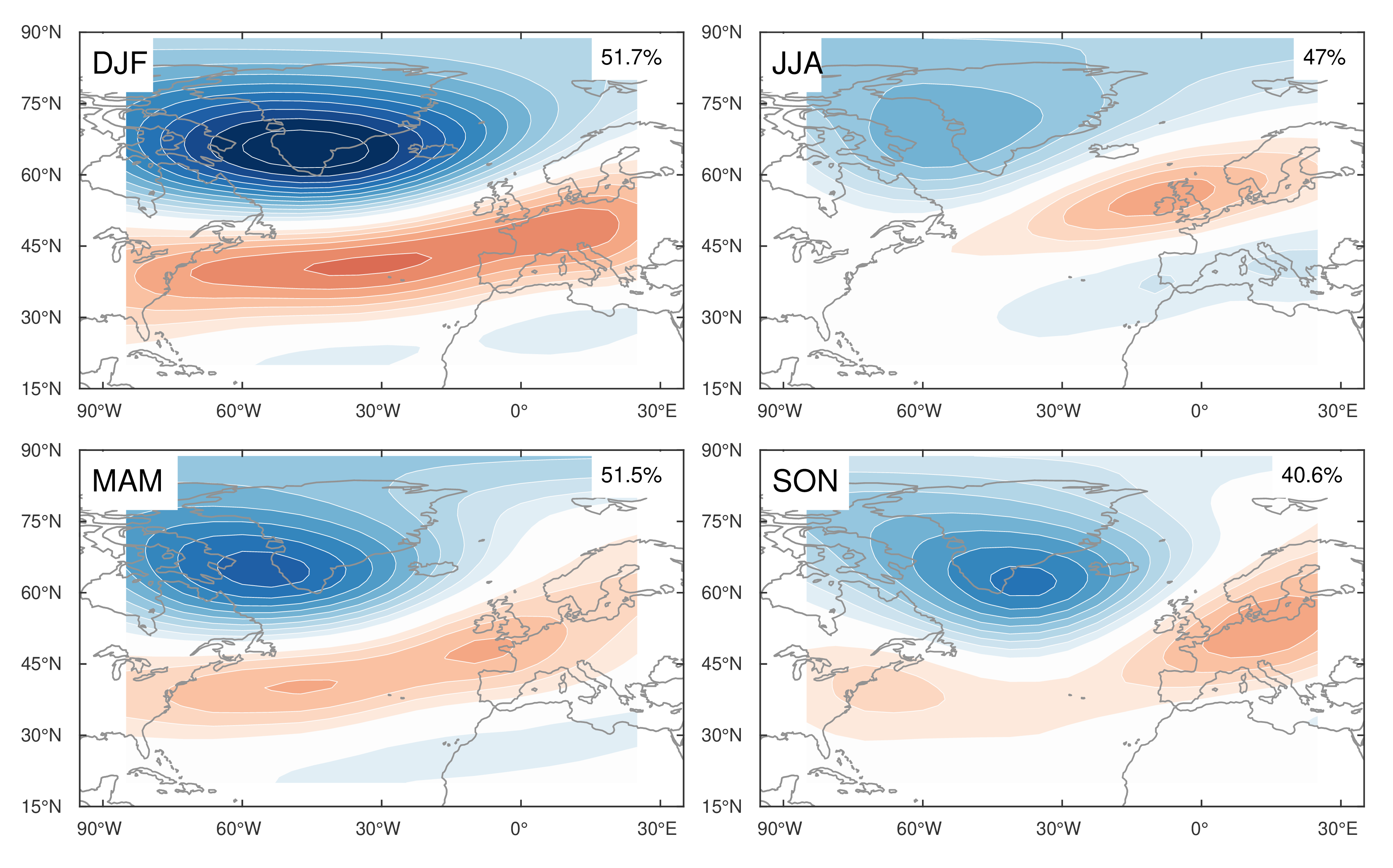
**

**Supplementary Figure 6: Seasonality of the NAO pattern**

NAO patterns obtained as the first EOF of 500 hPa height anomalies over the Euro-Atlantic sector are shown for the EOF analysis carried separately for the months of December-January-February (DJF), June-July-August (JJA), March-April-May (MAM) and September-October-November (SON). The contour interval is 10 m. The patterns shown correspond to one standard deviation of the associated time series. Red and blue are used for positive and negative values, respectively. The variance explained by the EOFs is indicated in each panel.

**
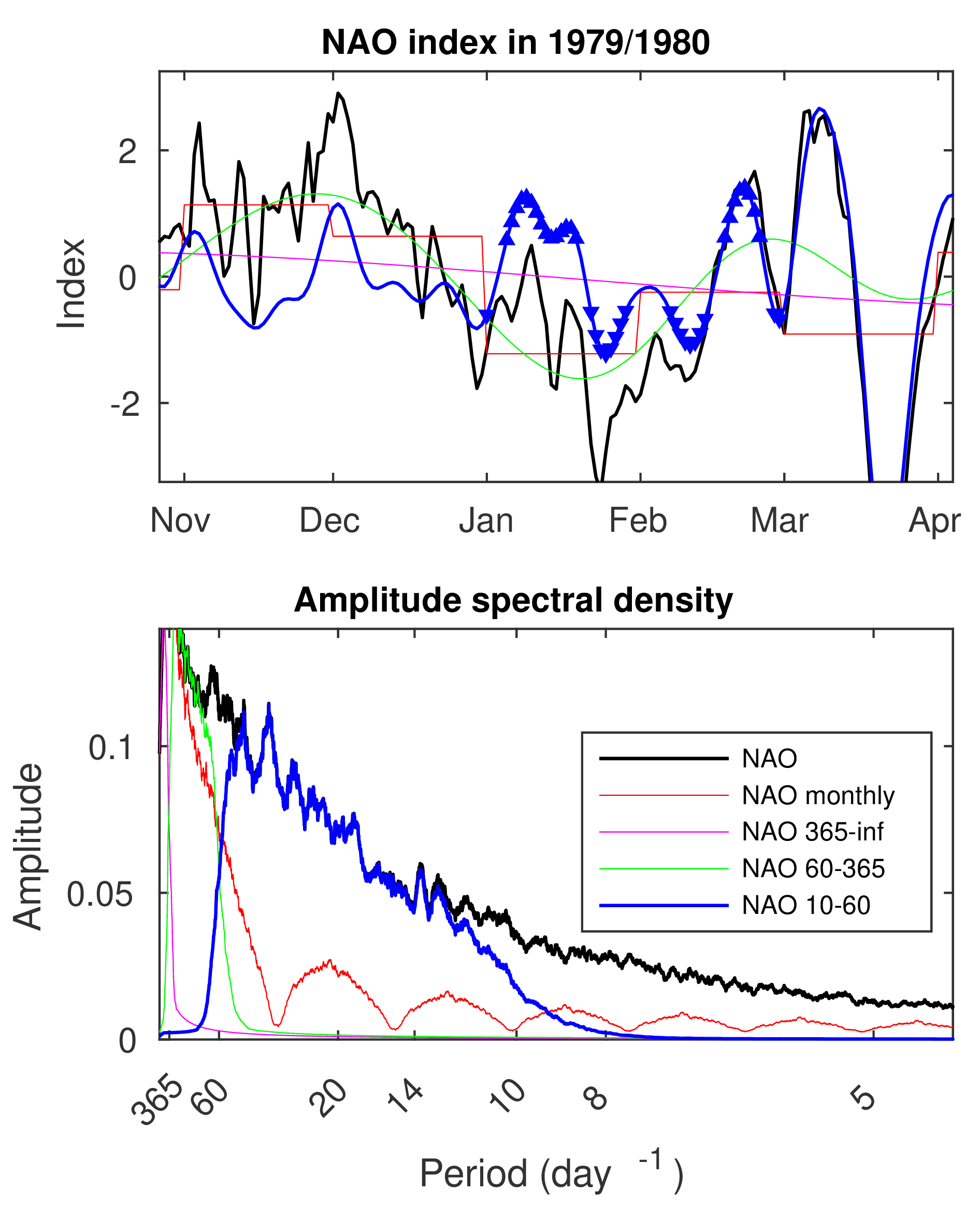
**

**Supplementary Figure 7: Illustrating the various time scales of NAO considered**

(Top) The NAO index is shown from August 1979 to June 1980. The original NAO index is shown in black and various time series resulting from applying time filtering to the original are shown in color according to the frequency band indicated in the legend. The dates used to define the positive and negative phases of NAO in DJF with the subseasonal component of NAO (blue, 10-60 days) are illustrated with upward pointing and downward pointing triangles, respectively. (Bottom) Amplitude spectral density of various frequency components of the NAO index computed from the continuous time series from 1958-2016. The spectral density is smoothed with a 51-point window for visualization purpose.


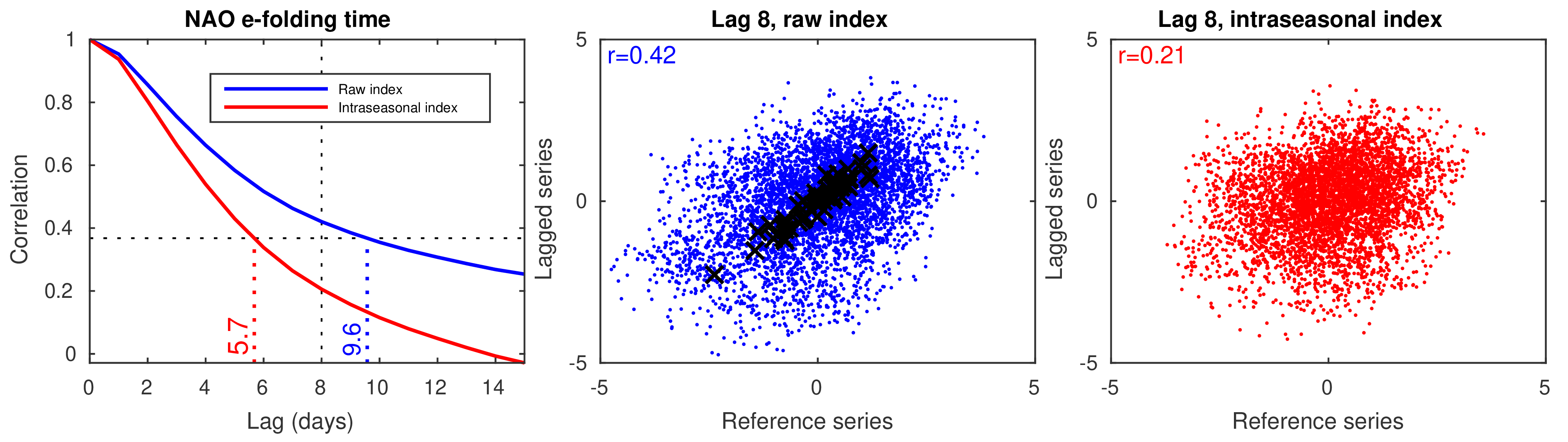


**Supplementary Figure 8: Assessing the *e*-folding time of the NAO index**

The *e*-folding time of the intraseasonal component of the NAO index is contrasted to the *e*-folding time of the raw index. The lag correlation (left) is compared between the two methods. The *e*-folding time is illustrated with vertical dotted lines. The correlation for the lag of 8 days is illustrated in scatter plots for (center) the raw NAO index and (right) the intraseasonal NAO index. Seasonal means are superimposed with black Xs on the scatterplot of the raw index, to demonstrate how interannual variability contributes to enhance the correlation.
